# Fukushima-derived radiocesium in the western North Pacific in 2014

**DOI:** 10.1007/s10967-016-5055-3

**Published:** 2016-10-03

**Authors:** Yuichiro Kumamoto, Michio Aoyama, Yasunori Hamajima, Hisao Nagai, Takeyasu Yamagata, Yoshimi Kawai, Eitarou Oka, Atsushi Yamaguchi, Keiri Imai, Akihiko Murata

**Affiliations:** 1 Research and Development Center for Global Change, Japan Agency for Marine-Earth Science and Technology, 2-15 Natushima-cho, Yokosuka, Kanagawa 237-0061 Japan; 2 Institute of Environmental Radioactivity, Fukushima University, 1-1 Kanayagawa, Fukushima, Fukushima 960-1296 Japan; 3 Low Level Radioactivity Laboratory, Kanazawa University, Wake, Nomi, Ishikawa 923-1224 Japan; 4 Department of Chemistry, College of Humanities and Sciences, Nihon University, 3-25-40 Sakurajousui, Setagaya Ward, Tokyo, 156-8550 Japan; 5 Atmosphere and Ocean Research Institute, The University of Tokyo, 5-1-5 Kashiwanoha, Kashiwa, Chiba 277-8564 Japan; 6 Graduate School of Fisheries Sciences, Hokkaido University, 3-1-1 Minato-cho, Hakodate, Hokkaido 041-8611 Japan; 7 School of Fisheries Sciences, Hokkaido University, 3-1-1 Minato-cho, Hakodate, Hokkaido 041-8611 Japan

**Keywords:** Fukushima Dai-ichi nuclear power plant accident, Radiocesium, North Pacific Ocean

## Abstract

In 2014, we measured activity concentration of radiocesium in the western North Pacific Ocean. In the north of Kuroshio Front high activity concentration of Fukushima-derived radiocesium in surface mixed layer in 2012 had been transported eastward by 2014. In the south of the front we found a radiocesium subsurface maximum in 200–600 m depth, which was similar to that observed in 2012. The subsurface maximum spread southward from 18°N to 15°N between 2012 and 2014, which suggests spreading of Fukushima-derived radiocesium into the whole western subtropical area by 2014 due to formation and subduction of the subtropical mode water.

**Electronic supplementary material:**

The online version of this article (doi:10.1007/s10967-016-5055-3) contains supplementary material, which is available to authorized users.

## Introduction

The massive Tohoku earthquake and consequent giant tsunamis on 11 March 2011 resulted in serious damage to the Fukushima Dai-ichi nuclear power plant (FNPP1) in eastern Japan [1]. Radiocesium (^134^Cs and ^137^Cs) released from the damaged FNPP1 caused radioactive contamination of the islands of Japan and the North Pacific Ocean mostly in March and April 2011 [2]. Measurements of ^134^Cs and ^137^Cs activity concentrations in soil collected in Japan revealed that (1) the activities of ^134^Cs and ^137^Cs released from FNPP1 were equivalent at a 1:1 ratio approximately [3] and (2) the total deposition of ^134^Cs (or ^137^Cs) on the land was 2.4 PBq (10^15^ Bq) [4]. On the other hand, the total deposition of ^134^Cs (or ^137^Cs) in the ocean had been estimated widely to be 9–37 PBq because of limited data obtained in the ocean [4–12]. In a recent study, observational data in surface water from commercial ships between April and June 2011 revealed that Fukushima-derived radiocesium deposited mainly in the north of the Kuroshio Front (30°N–35°N approximately), namely the subarctic area and a transition zone between the subarctic and subtropical areas [13]. Then the total deposition of ^134^Cs (or ^137^Cs) on the North Pacific was calculated more narrowly to be 12–15 PBq [14–16], suggesting more than 80 % of the atmospheric-released radiocesium was deposited on the North Pacific Ocean. ^134^Cs (or ^137^Cs) was also discharged directly into the ocean due to leakage of contaminated water from FNPP1 mainly in April 2011, which was estimated to be 2–6 PBq [1, 6, 8, 17–19]. However, temporal or spatial extrapolation in the calculation resulted in a larger amount of ^134^Cs (or ^137^Cs) from 11 to 27 PBq [20–22]. The smaller amount of directly-discharged radiocesium indicates that the total amount of Fukushima-derived ^134^Cs (or ^137^Cs) in the North Pacific Ocean was 14–21 PBq.

Radiocesium was also released into the open ocean before the FNPP1 accident by atmospheric nuclear weapons testing mainly in the 1950s and 1960s, nuclear fuel reprocessing mainly in the 1980s [23], and the Chernobyl accident in 1986. In the North Pacific Ocean the major source of radiocesium was atmospheric deposition due to the nuclear weapons testing [24]. The bomb-derived ^137^Cs deposited on the North Pacific was still there before the FNPP1 accident (1.0–2.5 Bq m^−3^ in surface seawater in the 2000s) [25] because its half-life is long, 30.17 y. In addition measurement of ^137^Cs in the North Pacific Ocean before the FNPP1 accident revealed that the bomb-derived ^137^Cs has been accumulated in the mid latitude, subtropical area [26]. After the accident, the Fukushima-derived ^137^Cs was added to the bomb-derived ^137^Cs, which was estimated to be about 30 % of the bomb-derived ^137^Cs [14]. In contrast, the ^134^Cs released before the accident had disappeared, because its half-life is only 2.06 y. Therefore ^134^Cs is a unique tracer for the FNPP1 accident and employed in this study in order to discuss temporal and spatial changes in the Fukushima-derived radiocesium.

Fukushima-derived ^134^Cs directly-discharged and atmospheric-deposited in the North Pacific Ocean was transported eastward along surface currents. In summer 2012, about one and half years after the accident, a water mass with high activity concentration of ^134^Cs (>5 Bq m^−3^) due to the direct discharge from FNPP1 (37.4°N/141°E) and deposition closed to it was observed in the central North Pacific around 165°E–170°W between 40°N and 50°N in surface seawater [27]. This high-^134^Cs surface water then shifted northward gradually and reached to stations in the Gulf of Alaskan in summer 2014 [28, 29]. A zonal eastward speed of this radioactive water mass was estimated to be about 4–8 cm s^−1^ [30].

In the south of the Kuroshio Front, namely the subtropical area, the activity concentration of ^134^Cs in surface water was lower than that in the north of the front (the transition zone and subarctic area), where the Fukushima-derived radiocesium was discharged and deposited, because the Kuroshio Front restricted surface water exchange across it [27]. In the western subtropical region, however, subsurface maxima of ^134^Cs (>10 Bq m^−3^) in approximately 200–600 m depth had been observed since about 10 months after the accident [29–33]. The subsurface layer of the ^134^Cs maxima correspond to density layers of subtropical mode water (STMW) [34] and central mode water (CMW) [35] in the North Pacific Ocean. Therefore it was concluded that the Fukushima-derived ^134^Cs was not only transported eastward in surface but also conveyed southward through subsurface due to formation and subduction of the mode waters.

Potential water density anomaly, *σ*_θ_ (kg m^−3^) defined by [potential water density—1000] of STWM ranges about 25.0–25.6 kg m^−3^. STMW is formed in just south of the Kuroshio/Kuroshio Extension current in the mid-winter due to severe cooling by cold monsoon wind and then transported southward through subsurface [36]. Therefore the main source of ^134^Cs observed in the STMW density was probably ^134^Cs deposited on the formation area just after the accident, in late March 2011. The total inventory of ^134^Cs decay-corrected to the accident date in STWM in 2012 was estimated to be about 4 PBq [33], which is 20–30 % of the total amount of ^134^Cs released in the North Pacific Ocean (14–21 PBq). CMW is denser than STMW (*σ*_θ_ = 26.0–26.6 kg m^−3^) and formed in the north of the Kuroshio Front [35]. However, mechanism of ^134^Cs entrainment into the CMW density is not clear because specific formation process of CMW has not been revealed yet. In addition, the total inventory of ^134^Cs in CMW is still unknown because the observational data are insufficient.

In 2014 we measured vertical profiles of radiocesium at 14 stations in the western North Pacific Ocean from the subarctic to subtropical areas (41°N–15°N) and discussed its temporal change between 2012 and 2014. As mentioned above temporal change in the Fukushima-derived radiocesium in surface layer in the north of Kuroshio Front has been roughly elucidated because there is enough data in surface water and the surface currents system in the North Pacific has been well understood. However, its temporal change in the mode waters in subsurface layer is still unknown due to limited number of vertical profile data. We compiled radiocesium data from this study and previous works and synthesized spreading of the Fukushima-derived radiocesium in the North Pacific Ocean by 2014.

## Experimental

### Samples

**Fig. 1:**
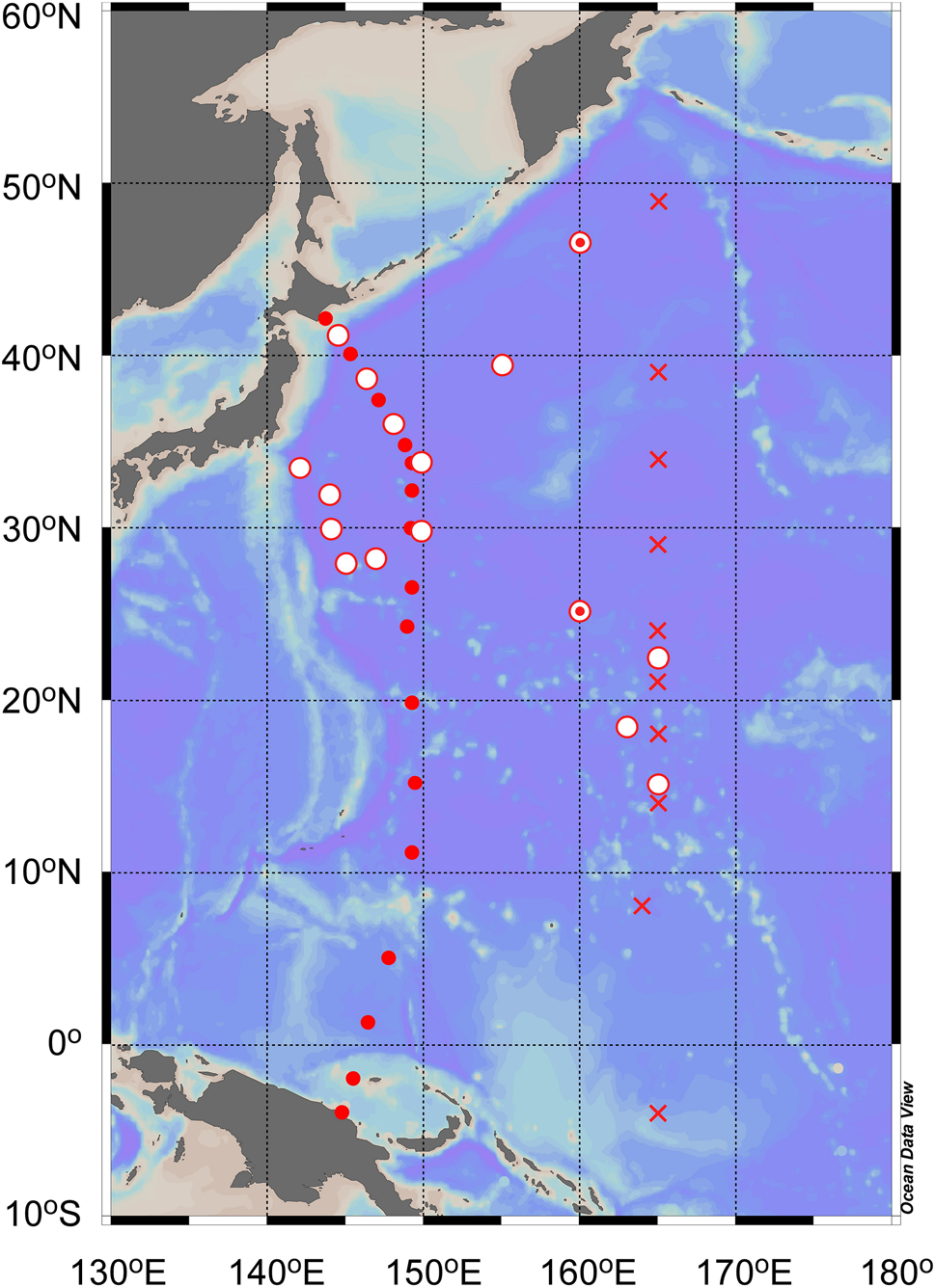
Sampling stations of seawater for radiocesium measurement in 2014. *Open circles* and *open circles* with *center dot* denote stations for a deep hydrocast to a depth of 800 m and for surface sampling only, respectively. Water sampling stations in previous studies conducted in January–February 2012 (*closed circles*, [41]) and June–July 2012 (*crosses*, [30]) are also shown. The map was drawn using Ocean Data View software [43]

Seawater samples for radiocesium measurements were collected at 16 stations between March and December 2014 during research cruises of KH14-01, OS-269, KS14-07, KH14-02, KY14-09, MR14-04, and KH14-06 in the western North Pacific Ocean (Fig. 1). Volume of seawater for each sample was about 20 or 40 l. Surface seawater was collected using a bucket, a 12 l niskin bottle (Model 1010X NISKIN-X, General Oceanics Inc.), or a pump for surface water from about several meter depth from surface. At 11 of all the 16 stations, seawater samples from deep layers (−800 m depth) were collected using the niskin bottles equipped to a carousel multi sampling system with sensors (Model SBE 9 plus/11 plus, Seabird Electronics Inc.) which measures conductivity (salinity), temperature, and depth. At three stations during the KH14-06 cruise the deep seawater was collected using a large volume water sampling system (LVWSS). LVWSS is equipped with four of 250 l sampling bottles, sensors, acoustic unit, and battery unit (model N12-1000, Nichiyu-Giken-Kohgyo Co. Ltd.). The seawater samples from the KH14-06 cruise were filtered using a polypropylene wound cartridge filter on board while those from the other cruises were not filtered. Ramzaev et al. [37] reported that radiocesium activity concentration in suspended particles was negligible relative to that in seawater in the western North Pacific even in 2012. Therefore we assume that activity determined in this study is derived from dissolved radiocesium in the solvent (seawater). All the samples were acidified by adding concentrated nitric acid.

### Measurements

After the cruises, radiocesium in the seawater sample was concentrated onto ammonium phosphomolybdate (AMP) for measurement of gamma-ray activity [38] in onshore laboratories of the Mutsu Institute for Oceanography at the Japan Agency for Marine-Earth Science and Technology (MIO/JAMSTEC), General Environmental Technos Co., Ltd., or Nihon University (NU). The radiocesium activity in the AMP/Cs compounds was measured using low-background Ge-detectors in Ogoya underground laboratory of the Low Level Radioactivity Laboratory at Kanazawa University (LLRL/KU) [39], MIO/JAMSTEC, or NU. The average of analytical uncertainty (one standard deviation or 68 % confidence level) for ^134^Cs and ^137^Cs measurements were 10–30 and 6–9 %, respectively. Detection limit for ^134^Cs/^137^Cs decay-corrected to the accident date (11 March 2011) for the measurement at LLRL/KU, MIO/JAMSTEC, and NU were calculated to be about 0.2/0.05, 0.4/0.1, and 0.7/0.2 Bq m^−3^, respectively. The detection limits of ^134^Cs measurement for samples from the KH14-06 were larger than the others because those measuring time were shorter. In MIO/JAMSTEC, the Ge-detectors were calibrated with gamma-ray volume sources (Eckert & Ziegler Isotope Products) certificated by Deutscher Kalibrierdienst (DKD). Measurements of ^137^Cs activity in AMP/Cs compounds derived from certified reference materials (IAEA-443 [40]) among the three laboratories resulted in good agreement within the uncertainty of the certificated value, which confirmed the comparability of the radiocesium measurements at the three laboratories.

## Results and discussion

### Vertical distribution of radiocesium in 2014

**Fig. 2:**
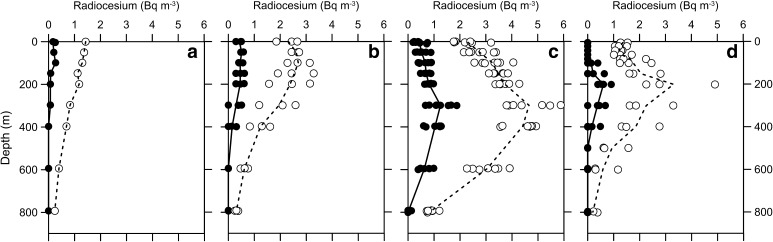
Vertical profiles of activity concentrations (Bq m^−3^) of ^134^Cs (*closed circles*) and ^137^Cs (*open circles*) at a station(s) in the north of 40°N (**a**), between 35°N and 40°N (**b**), between 25°N and 35°N (**c**), and between 15°N and 25°N (**d**) in the western North Pacific in 2014. *Solid* and *broken lines* indicate averaged activity concentrations of ^134^Cs and ^137^Cs at each sampling depth, respectively. The activity concentration was corrected to the sampling date

Figure 2 shows vertical profiles of activity concentration of radiocesium (Bq m^−3^) in the western North Pacific in 2014. The data were grouped into four by latitude; data in the north of 40°N (Fig. 2a), between 35°N and 40°N (Fig. 2b), between 25°N and 35°N (Fig. 2c), and between 15°N and 25°N (Fig. 2d). These areas approximately correspond to the subarctic area, the transition zone, and the northern and southern subtropical areas, respectively. The subarctic (subtropical) area and transition zone is divided by the Subarctic (Kuroshio) Front. The vertical profiles of ^134^Cs and ^137^Cs are similar in each area. The activity concentration of ^134^Cs, however, was lower than that of ^137^Cs because of its shorter half-life (2.06 y) and the pre-existing ^137^Cs derived from the nuclear weapons testing. In the transition zone (Fig. 2b) the activity concentration of ^134^Cs (0.5 Bq m^−3^ in average) was highest in the surface mixing layer above about 200 m depth. Below that depth the activity concentration decreased with depth and then lessened below the detection limit at 600 and 800 m depths. In the subtropical area, or the south of the Kuroshio Front (Fig. 2c, d) a subsurface maximum of ^134^Cs was observed at all the stations. The activity concentration of the maximum peak in the northern subtropical area (1.3 Bq m^−3^ in average) were higher than those in the southern subtropical area (0.6 Bq m^−3^ in average). Depths of the maximum peak in the north (about 300 m) were deeper than those in the south (about 200 m). The activity concentration in the surface mixing layer in the northern subtropical areas (0.6 Bq m^−3^ in average) was higher than that in the north of the front, which is opposite to results obtained in 2012. Except that, the distribution of radiocesium in 2014 was similar with that observed in 2012 [30, 41] although the activity concentration in 2014 was lower than that in 2012 as mentioned below.

### Temporal change in vertical distribution

**Fig. 3:**
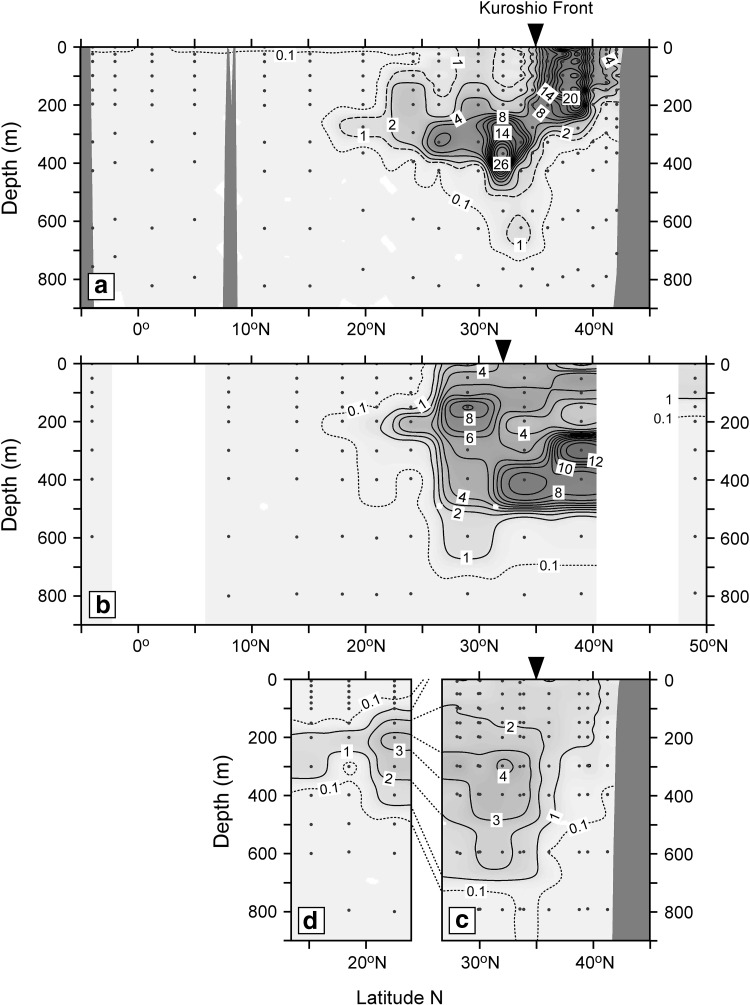
Cross sectional views of ^134^Cs activity concentration (Bq m^−3^) along approximately 145°E–150°E in January–February 2012 (**a**) [41], 165°E in June–July 2012 (**b**) [30], approximately 142°E–155°E in March–July 2014 (**c**), 163°E–165°E in December 2014 (**d**). The activity concentration of ^134^Cs was corrected to the accident date. Contour interval in **a** is 2 Bq m^−3^ except for broken (1 Bq m^−3^) and *dotted* (0.l Bq m^−3^) *lines*. Contour interval in **b**, **c**, and **d** is 1 Bq m^−3^ except for *dotted lines* (0.l Bq m^−3^). *Dots* show sampling depths at each station for radiocesium measurements. *Arrows* indicate approximate position of the Kuroshio Front. This figure was drawn using Ocean Data View software [43]

In order to discuss temporal change in the vertical distribution between 2012 and 2014, the activity concentration of ^134^Cs was corrected to the accident date for radioactive decay and plotted in sectional view along latitude (Fig. 3). In January–February 2012 along approximately 145°E–150°E (Fig. 3a) [41], high activity concentration in surface mixed layer above about 200 m depth (10 Bq m^−3^ in average) was observed in the north of the Kuroshio Front, which was mainly derived from eastward movement of ^134^Cs directly-discharged from FNPP1 and deposited closed to FNPP1 along surface currents. In the south of the front, there was a subsurface maximum around 300 m depth between 20°N and 35°N approximately. The water density anomaly of the subsurface maximum layer agreed with that of STMW, 25.0–25.6 kg m^−3^.

About a half year later a sectional view along 165°E was achieved (Fig. 3b) [30]. In the north of the Kuroshio Front, activity concentration of ^134^Cs decay-corrected to the accident date in the surface mixed layer along 165°E in June–July 2012 (3 Bq m^−3^ in average) was lower than that observed along 145°E–150°E in January–February 2012. By the summer of 2012, a main body of the ^134^Cs directly-discharged and deposited closed to FNPP1 had been transported to the central North Pacific [27]. Thereby this ^134^Cs decrease in the surface layer could be explained by dilution due to water mixing during the eastward transportation. Below the surface mixing layer there was a subsurface maximum between 300 and 500 m depth approximately, which was not observed along 145°E–150°E in January–February 2012 (Fig. 3a). The water density anomaly of this subsurface maximum layer ranged 26.0–26.6 kg m^−3^, which corresponds to that of CMW denser than STMW. CMW is formed in an area in the north of the Kuroshio Front and the east of 150°E in the mid-winter, transported eastward, and then southward through the subsurface layer [35]. The appearance of the subsurface maximum of ^134^Cs in the density layer of CMW along 165°E and not along 145°E–150°E suggests the formation area of CMW in the east of 150°E, where the Fukushima-derived ^134^Cs was subducted in the mid-winter (March) of 2011 and/or 2012. In the south of the front, another subsurface maximum around 200 m depth was observed. The water density anomaly of this shallower subsurface maximum ranged 25.0–25.6 kg m^−3^, which corresponds to that of STMW as same as that observed along 145°E–150°E in January–February 2012.

In March–July 2014 along approximately 142°E–155°E, the high concentration activity in the north of the Kuroshio Front observed in January–February 2012 disappeared (Fig. 3c). This is probably due to the eastward propagation of the contaminated surface water. A subsurface ^134^Cs maximum in the STWM density layer in the south of the front is similar to that observed in 2012, which contrasts to the disappearing of the high activity concentration of ^134^Cs in the surface mixing layer in the north of the front. STMW is transported eastward/southward and circulated within the western subtropical gyre although its horizontal speed is slower than that of the surface water current [36], which could result in persisting of the subsurface maximum of ^134^Cs in 2014. The activity concentration of the maximum peak around 300–400 m depth decreased by one-tenth at most and the peak shape was eroded vertically, which can be explained by entrainment into surface mixed layer and deep penetration due to vertical diffusion.

In December 2014 along 163°E–165°E between 15°N and 23°N, we also found a ^134^Cs maximum peak in subsurface layer of STWM (Fig. 3d). Although the activity concentration decay-corrected to the accident date of the maximum peak in 2014 at 23°N (about 3 Bq m^−3^) was almost equivalent with that observed in 2012, the subsurface maximum has been spread vertically from 100 to 400 m approximately in 2014. In addition the subsurface maximum spread southward from 18°N to 15°N at least between 2012 and 2014.

### Temporal change in vertical inventory

**Fig. 4:**
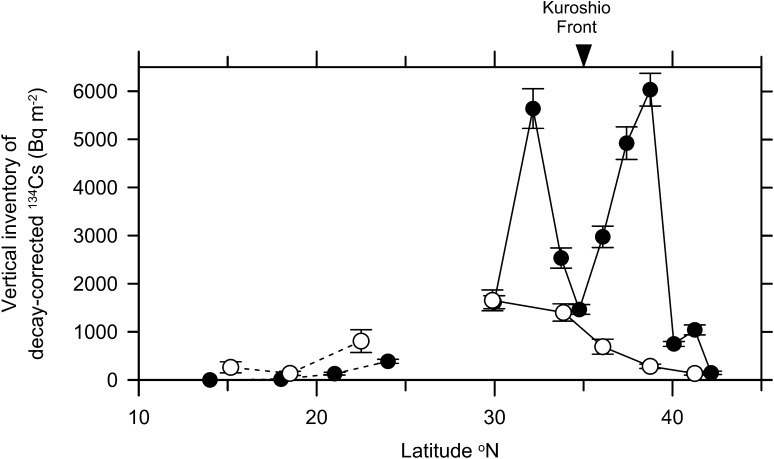
Vertical inventories of ^134^Cs (Bq m^−2^) in 2012 (*closed circles*) [30, 41] and 2014 (*open circles*) along 145°E–150°E (*solid lines*) and 163°E–165°E (*broken lines*). Data in 2014 are only from cruises of MR14-04 and KH14-06. The inventory of ^134^Cs was corrected to the accident date. An *arrow* indicates approximate position of the Kuroshio Front. *Error bars* indicate uncertainty (one standard deviation or 68 % confidence level) of the vertical inventory, about 10 %

**Fig. 5:**
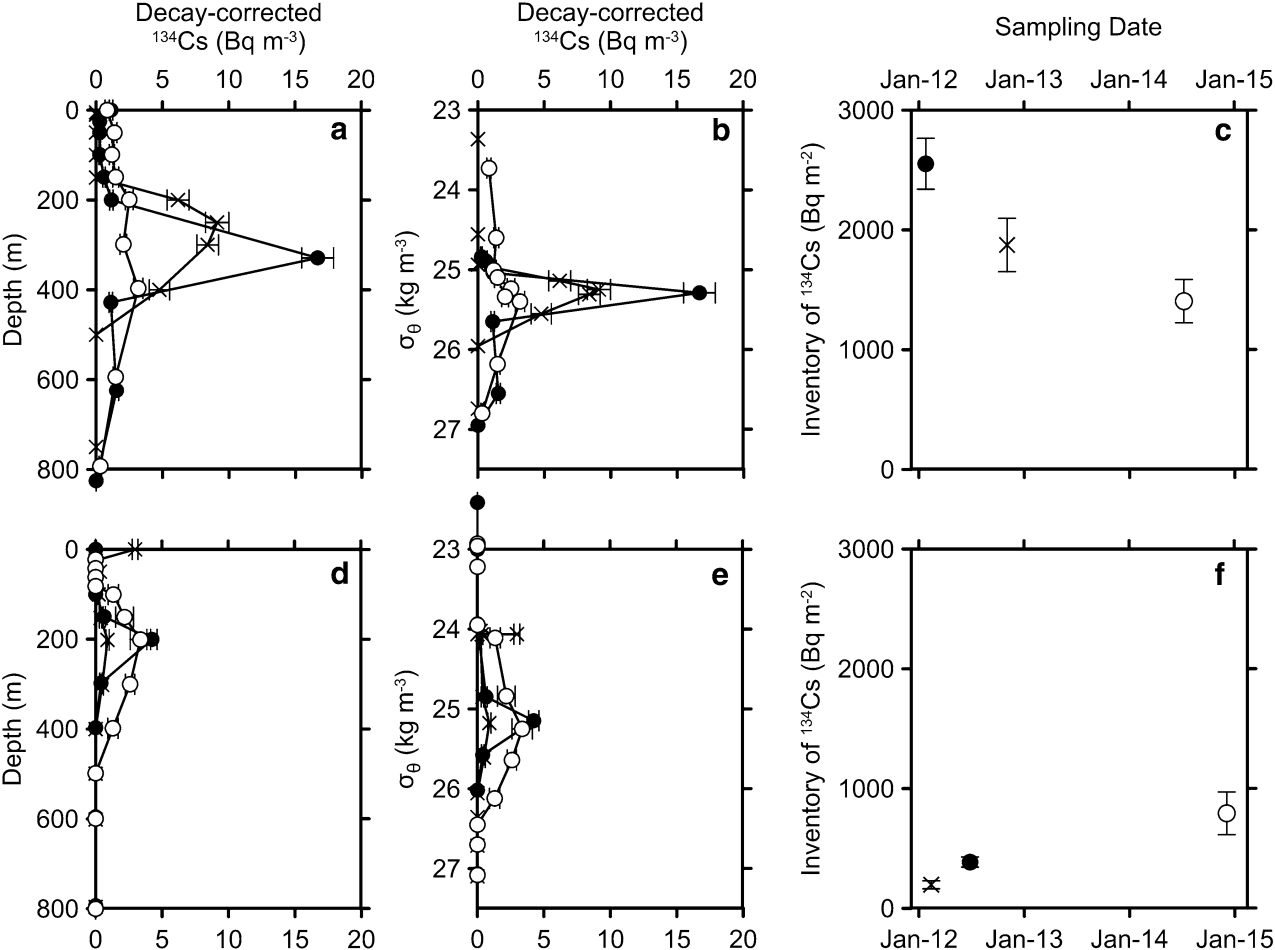
Activity concentrations of ^134^Cs (Bq m^−3^) against sampling depth (**a**) and water density anomaly, *σ*_θ_ (**b**), and temporal change in ^134^Cs vertical inventory from surface to 800 m depth (**c**, Bq m^−2^) at stations in 34°N/147–150°E in January 2012 (*closed circles* [41]), November 2012 (*crosses* [33]), and July 2014 (*open circles*). *Error bars* in **c** indicate uncertainty (one standard deviation or 68 % confidence level) of the vertical inventory, about 10 %. **d, e**, **f** are same as **a**, **b**, **c**, respectively, but for at stations in 23–24°N/165°E in February 2012 (*crosses* [30]), June 2012 (*closed circles* [30]), and December 2014 (*open circles*). The activity concentration and vertical inventory were corrected to 11 March 2011

Temporal change in vertical inventory of ^134^Cs decay-corrected to the accident date from surface to 800 m depth indicates that the Fukushima-derived ^134^Cs decreased (increased) in the north (south) of 30°N between 2012 and 2014 (Fig. 4). In the north of the Kuroshio Front, a large amount of ^134^Cs, up to 6000 Bq m^−2^ reduced to less 1000 Bq m^−2^ due to the eastward transportation along with the surface current. Between 30°N and the front (35°N) along 145°E–150°E the vertical inventory decreased because the activity concentration of maximum peak around 300 m depth decreased (Fig. 3). In addition to the observations in January–February 2012 and March–July 2014, a sectional view of ^134^Cs was also observed along 147°E between 30°N and 41°N in October–November 2012 and a subsurface maximum of ^134^Cs was also found in density layer of STMW in the south of 35°N [33]. At stations in 34°N/147–150°E, just south of the Kuroshio Front, ^134^Cs activity concentration decay-corrected to the accident date in the subsurface maximum peak was decreasing from January 2012 to July 2014 (Fig. 5a), which results in monotonous decrease in the vertical inventory (Fig. 5c).

At stations in the southern subtropical area between 15°N and 25°N along 163°E–165°E, the ^134^Cs vertical inventory increased thrice in average between 2012 and 2014 (Fig. 4) due to the vertical and horizontal (southward) spreading of its subsurface maximum. At stations in 23–24°N/165°E, ^134^Cs activity concentration decay-corrected to the accident date in the subsurface maximum peak around 200 m depth increased from 0.9 to 4.3 Bq m^−3^ between February 2012 and June 2012, and then decreased slightly in December 2014 to be 3.4 Bq m^−3^ (Fig. 5d). The vertical inventory, however, increased about twice between June 2012 and December 2014 (Fig. 5f) since the subsurface maximum spread vertically in 2014.

The subsurface maximum of Fukushima-derived ^134^Cs in the subtropical area appeared in the density layer of STMW, 25.0–25.6 kg m^−3^ *σ*_θ_ (Fig. 5b, e) although its peak depths in the northern subtropical area were deeper than those in the southern area (Fig. 5a, d). In density layer of CMW (26.0–26.6 kg m^−3^ *σ*_θ_), a subsurface maximum of ^134^Cs was not observed in 2014, which implies that the subsurface maximum in the CMW layer observed in the transition zone in June 2012 (Fig. 3b) was transported southward across the Kuroshio Front. Therefore the erosion of the subsurface peak by diapycnal mixing (mixing between waters with different density) could result in downward (and upward) spreading of ^134^Cs in 2014. In short, the vertical inventory at the northern stations (34°N) decreased due to the decrease in the activity concentration of the subsurface maximum peak while that at the southern stations (23°N–24°N) increased because of the vertical spreading of the peak. The vertical inventory at 30°N/149°E did not change apparently between 2012 and 2014 (Fig. 4) because the decrease in activity concentration of the peak balanced with the increase in vertical spreading of the peak. These results indicate that the Fukushima-derived radiocesium in the western subtropical area has been transported southward due to subduction and advection of STMW with the vertical spreading due to diapycnal mixing.

## Conclusions

**Fig. 6:**
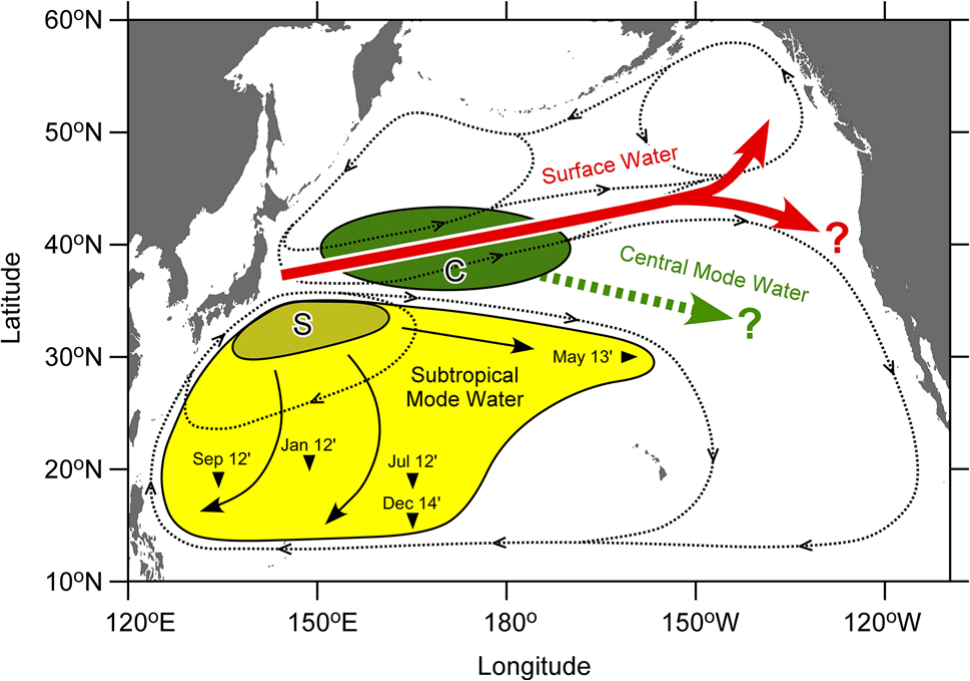
A schematic view of spreading of the Fukushima-derived radiocesium in the North Pacific Ocean by 2014. *Thick arrows* indicate pathways in surface mixed layer in the transition zone and subarctic area. A *thick broken arrow* is a speculated direction in subsurface layer of the central mode water (CMW, 26.0–26.6 *σ*_θ_). *Thin arrows* show spreading directions in subsurface layer of the subtropical mode water (STMW, 25.0–25.6 *σ*_θ_). An *open area* indicates approximate distribution of STMW derived from vorticity [44]. *Shaded areas* of “C” and “S” are formation areas of CMW and STMW, respectively [45]. *Small arrows* in the STMW area indicate observed southern/western edges of Fukushima-derived radiocesium spreading in the density layer of STMW [29, 30, 32, 41]. *Dotted lines* show surface water currents. The map in this figure were drawn using Ocean Data View software [43]

Figure 6 shows a schematic view of spreading of the Fukushima-derived radiocesium in the North Pacific Ocean by 2014, which is derived from results of this study and previous works. We measured radiocesium in the western North Pacific Ocean in 2014 and revealed its temporal change between 2012 and 2014. The high concentration activity observed in 2012 in the north of the Kuroshio Front disappeared in 2014. This is probably due to the eastward propagation along the surface current as reported in the previous works [27, 28, 30, 42]. Although the eastward surface current turns north and south in the eastern edge of the basin, southward transportation of the Fukushima-derived radiocesium off the west coast of the North American Continent has not been observed yet. We found that the Fukushima-derived radiocesium had reached to the southern edge of the western subtropical area, about 15°N by 2014. The Fukushima-derived radiocesium was also observed in the density layer of STMW at 30°N/160°W in May 2013 [29]. These results suggest that the Fukushima-derived radiocesium had spread into the whole western subtropical area by 2014 due to formation and subduction of STMW. STMW circulates within the western subtropical area. However, northward return of the Fukushima-derived radiocesium along the circulation has not been observed yet. A subsurface maximum in density layer of CMW was not observed in the south of the Kuroshio Front in 2014, implying that the subsurface maximum observed in the north of the front in June 2012 (Fig. 3b) had been transported eastward, not southward yet by 2014. Further observations for radiocesium measurement in the North Pacific Ocean are necessary to follow the spreading of the Fukushima-derived radiocesium in the basin.

## Electronic supplementary material

Below is the link to the electronic supplementary material.

Supplementary material 1 (XLSX 38 kb)
